# Detection of *NRAS* mutation in cell-free DNA biological fluids from patients with kaposiform lymphangiomatosis

**DOI:** 10.1186/s13023-019-1191-5

**Published:** 2019-09-11

**Authors:** Michio Ozeki, Yoko Aoki, Akifumi Nozawa, Shiho Yasue, Saori Endo, Yumiko Hori, Kentaro Matsuoka, Tetsuya Niihori, Ryo Funayama, Matsuyuki Shirota, Keiko Nakayama, Toshiyuki Fukao

**Affiliations:** 1 Department of Pediatrics, Graduate School of Medicine, Gifu University, Yanagido 1-1, Gifu, 501-1194 Japan; 2 Department of Medical Genetics, Tohoku University School of Medicine, Sendai, 980-8574 Japan; 3 Department of Pathology, Graduate School of Medicine, Osaka University, Osaka, 565-0871 Japan; 4 Department of Pathology, Dokkyo Medical University Saitama Medical Center, Saitama, 343-8555 Japan; 5 Division of Cell Proliferation, United Centers for Advanced Research and Translational Medicine, Tohoku University Graduate School of Medicine, Sendai, 980-8575 Japan; 6 Division of Interdisciplinary Medical Science, United Centers for Advanced Research and Translational Medicine, Tohoku University Graduate School of Medicine, Sendai, 980-8575 Japan

**Keywords:** Vascular anomaly, Kaposiform lymphangiomatosis, Neuroblastoma RAS viral oncogene homolog, Cell-free DNA, Liquid biopsy

## Abstract

**Background:**

Kaposiform lymphangiomatosis (KLA) has recently been distinguished as a novel subtype of generalized lymphatic anomaly (GLA) with foci of spindle endothelial cells. All cases of KLA involve multiple organs and have an unfavorable prognosis. However, the molecular pathogenesis is unknown, and there are no useful biomarkers. In the present study, we performed genetic analysis to elucidate the cause of this disease and detect biomarkers for it.

**Methods:**

We performed whole-exome sequencing of DNA samples from leukocytes and a biopsy specimen and analyzed cell-free DNA (cfDNA) from plasma and pleural effusion of patients to identify the *NRAS* c.182A > G (p.Q61R) mutation using the droplet digital polymerase chain reaction (ddPCR).

**Results:**

All KLA patients (patients 1–5) had invasive and aggressive features (hemorrhagic pleural effusions, coagulation disorder, and thrombocytopenia) and characteristic findings of KLA in their pathological examinations. In whole exome sequencing for patient 1, c.182A > G missense variant (p.Q61R) in *NRAS* was identified in fresh frozen samples of a mass on the left chest wall at a frequency of 5% of total alleles but not in his blood leukocytes. Furthermore, the same mutation was detected in cfDNA isolated from plasma and pleural effusion by using ddPCR. ddPCR analysis of plasma/pleural effusion samples from an additional four KLA patients showed that the same mutation was detected in isolated cfDNA in three of the four, as well as in a tissue sample from one of the three plasma/effusion-positive patients that had been obtained to confirm the mutation.

**Conclusion:**

These results provide the first evidence that *NRAS* oncogenic variant was identified in DNA samples from KLA patients from not only two affected lesions but also plasma and pleural effusion.

## Background

Lymphatic anomalies (LAs), including generalized lymphatic anomaly (GLA) and kaposiform lymphangiomatosis (KLA), are extremely rare diseases with severe symptoms and poor prognosis [1]. KLA is categorized as a novel subtype of GLA in the International Society for the Study of Vascular Anomalies (ISSVA) classification updated in 2018 [2]. It is described as an aggressive disease of the lymphatic system and has foci of “kaposiform” abnormal spindle lymphatic endothelial cells; however, the pathogenesis of the patients remains unknown [3].

Recently, genetic research has attempted to elucidate the actual conditions and pathogenesis of vascular anomalies [4]. Low-level somatic mutations of PI-3 kinase or the RAS pathway have been detected in samples from vascular anomalies [4]. In recent studies, the *NRAS* c.182A > G (p.Q61R) mutation was detected in affected lesions of KLA and GLA patients [5, 6]. However, a tissue biopsy can lead to severe complications, such as bleeding or lymphatic leakage. Therefore, development of noninvasive diagnostic methods may not only provide a more accurate diagnosis, but also decrease the risk of complications. Analysis of cell-free DNA (cfDNA), so-called liquid biopsy, is becoming a promising clinical application for molecular testing and cancer detection [7].

Here, we describe a case of KLA, in which a somatic mutation in the *NRAS* gene was detected both in a biopsy specimen and in cfDNA. Additionally, the same mutation was detected in cfDNA from the four other patients with KLA and from a tissue sample obtained from one of them. Our data therefore suggest that cfDNA analysis might be a useful method for diagnosing KLA.

## Methods

### Patients

Medical records from January 2016 to December 2018 on 8 patients (5 with KLA and 3 with GLA) were retrospectively analyzed. This study was approved by the Ethics Committees of Gifu University School of Medicine and Tohoku University School of Medicine (25–136 and 2013–1–278). The patients or their legal guardians gave their informed consent to be included. Data on patient characteristics, diagnosis, clinical symptoms, and test results were collected.

### Genetic analysis of a patient with KLA (patient 1)

Genomic DNA was extracted from blood samples using standard protocols. DNA was extracted from frozen samples of a mass on the left chest wall of patient 1 using Qiagen DNeasy Blood and Tissue Kit.

In whole-exome sequencing (WES), targeted enrichment was performed using the SureSelect Human All Exon V6 kit. Exon-enriched DNA libraries were sequenced on the Illumina Hiseq 2500 for 101 bp. Burrows–Wheeler Alignment (BWA) was used to align the sequence reads to the human genome (hg19); all parameters of BWA were kept at the default settings. Following removal of duplicates from the alignments, realignment around known indels, recalibration, and SNP/indel calling were performed with the UnifiedGenotyper tool of the Genome Analysis Toolkit (GATK) (1.5) [8]. ANNOVAR was used for the annotation against the RefSeq database and dbSNP [9]. Novel mutations were extracted according to the variants located in an exon or splice site, excluding synonymous variants, and variants that exhibited an allele frequency of less than 1% or were not reported in variant databases in the 1000 Genomes Project (http://browser.1000genomes.org/), the Exome Aggregation Consortium (ExAC, http://exac.broadinstitute.org/), the genome aggregation database (gnomAD, https://gnomad.broadinstitute.org/), the Human Genome Variation Database (HGVD, http://www.genome.med.kyoto-u.ac.jp/SnpDB/), and the Integrative Japanese Genome Variation Database (iJGVD, https://ijgvd.megabank.tohoku.ac.jp/). Detected variants were confirmed through visual examination of the genetic data using the Integrative Genomics Viewer (IGV; http://www.broadinstitute.org/igv/).

To identify low-frequency somatic mutations in frozen samples of a mass on the left chest wall, variants were called by MuTect. Identified variations were visually reviewed by IGV.

PCR products of exon 2 in *NRAS* were subcloned using a pTOPO TA cloning kit (Invitrogen, Carlsbad, CA) and transformed in TOP10F competent cells (Invitrogen). Plasmids were purified from each colony and sequenced.

### Droplet digital polymerase chain reaction (ddPCR) analysis on *NRAS* p.Q61R mutation

CfDNA was extracted using a QIAamp Circulating Nucleic Acid Kit (Qiagen). Extracted DNA was quantified using a Qubit® 3.0 Fluorometer (Thermo Fisher Scientific). Genomic DNA was extracted from formalin-fixed, paraffin-embedded (FFPE) tissue using a Maxwell RSC DNA FFPE Kit (Promega). *NRAS* mutation detection assays were performed using the mutation detection assay *NRAS* p.Q61R c.182A > G (Bio-Rad) in a ddPCR apparatus (QX200™ AutoDG™ Droplet Digital™ PCR system; Bio-Rad). Each ddPCR assay was performed in triplicate for each sample (patients 1–8). We used Bio-Rad QuantaSoft Analysis Pro for data analysis. The data are expressed as a percentage of mutant droplets relative to the sum of wild-type droplets of each sample. The experiment featured a positive control, which included only wild-type DNA, and a negative control, which did not include DNA. The genome of the human skin melanoma cell line (SK-MEL-2) was used as a positive control as it is known to have an *NRAS* Q61R mutation.

## Results

### Case description

#### Patient 1 (P1)

**Fig. 1:**
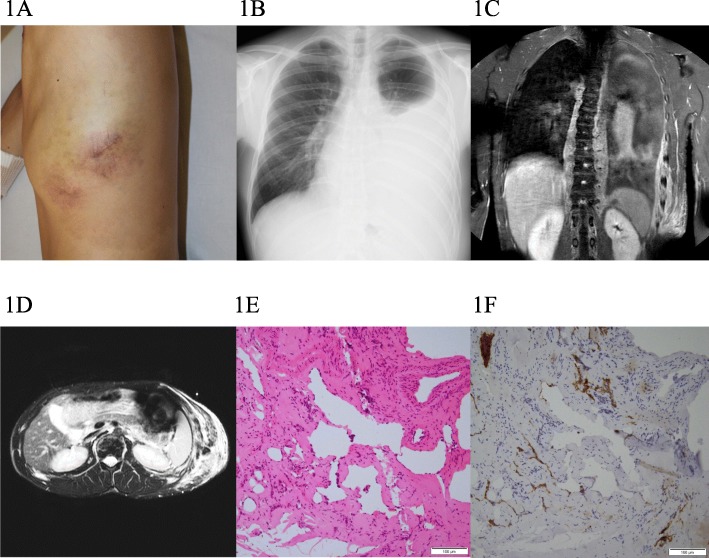
Clinical appearance and examinations of the patient with KLA (P1). **a** Cutaneous manifestation of left chest wall. **b** Chest radiography shows pleural effusion and mediastinal enlargement. **c**, **d** Chest contrast-enhanced MRI demonstrates diffuse thickening of the left chest wall, pleural effusion in the left lung, and retroperitoneal soft-tissue mass. **e**, **f** Specimen shows proliferation of thin-walled, anastomosing lymphatic vessels lined by a single layer of endothelial cells with a focus of spindle cells (bar 100 μm, H&E). Endothelial cells were identified as lymphatics using D2–40 (bar 100 μm)

A 19-year-old male suffered from hemoptysis, dyspnea, and gastrointestinal hemorrhage. He had had a subcutaneous mass at the left chest wall from birth. The patient had previously been admitted to another hospital for examination and management. The examination revealed pleural effusion, multiple osteolytic lesions of the spine, and a splenic cystic lesion. The patient had been diagnosed with lymphangiomatosis and treated with conservative therapy; however, his symptoms did not improve. Two years later, he was therefore transferred to Gifu University Hospital for alternative treatment. Physical examination revealed a reddish brown, soft, and nontender subcutaneous mass (10 × 8 cm) on the left chest wall with no signs of inflammation (Fig. 1a). Plain radiography and gadolinium-enhanced magnetic resonance imaging (MRI) of the chest revealed pleural effusion, an infiltrative soft-tissue mass at the left chest wall, and a retroperitoneal lesion (Fig. 1b–d). Laboratory data indicated mild thrombocytopenia (9.0 × 10^4^/μL) and coagulopathy (D-dimer: 31.0 μg/ml, fibrinogen: 181 mg/dL (reference range for D-dimer is < 0.5 μg/ml and for fibrinogen 200–400 mg/dL). Colonoscopy showed bleeding and multiple dilated tortuous blood vessels running along the surface of the sigmoid colon. An open biopsy specimen of the subcutaneous lesion showed irregularly dilated endothelial cells and foci of spindle cells (Fig. 1e). In immunohistochemical study, both endothelial cells and spindle cells showed positive reactivity for CD31, D2–40, and Prox-1, identifying them as being of lymphatic origin (Fig. 1f). Following these results, the diagnosis of KLA was made. We decided to undertake treatment using a mammalian target of rapamycin (mTOR) inhibitor, sirolimus. The treatment was approved by the review board of Gifu University Hospital and written informed consent was obtained from the patient. Sirolimus treatment was started at 2 mg/day. Dose adjustments were made to maintain the drug at a therapeutic level of 5 to 15 ng/ml. Within 2 weeks of starting the sirolimus treatment, pleural effusion and gastrointestinal hemorrhage decreased and the lesions of the chest wall shrank. After 6 months, the coagulopathy improved (D-dimer: 11.2 μg/ml, fibrinogen: 260 mg/dL); however, the mild thrombocytopenia did not change. At the time of writing, the patient has been continuing therapy for 2 years without any adverse effects.

#### Patients 2–8 (P2–8)

Patients 2, 3, 4, and 5 (P2–5) were diagnosed with KLA based on clinical and pathological findings (Table 1). All patients had invasive and aggressive features and characteristic findings of KLA (foci of spindle cells) in their pathological examinations. In contrast, patients 6, 7, and 8 (P6–8) were diagnosed with GLA because they did not have characteristic evidence of KLA. Only one patient in the P2–8 group (P2) had undergone genetic analysis of the affected lesions because we did not have fresh frozen samples from the others (P3–8).

**Table 1:** Patient characteristics and results from a droplet digital PCR experiment on *NRAS* p.Q61R mutation

Patient number (diagnosis)	Age (years) / sex	Affected organs	Complications	Sample material (sample numbers in Fig. 3b)	Target gene	Concentration (copies/μL)^a^	Accepted droplets^b^	Fractional abundance (%)^c^	Poisson fractional abundance (max)^d^	Poisson fractional abundance (min)^e^
P1 (KLA)	21/ M	Chest wall and mediastinum	Hemorrhagic PE, gastrointestinal hemorrhage and coagulation disorder	Plasma (P1–1)	*NRAS* Q61R	0.41	40,029	0.18	0.28	0.08
					*NRAS* WT	227.07	40,029	–	–	–
				PE (P1–2)	*NRAS* Q61R	0.41	31,317	0.44	0.70	0.18
					*NRAS* WT	93.64	31,317	–	–	–
P2 (KLA)	12/ M	Bone, thoracic and mediastinal	Hemorrhagic PE and coagulation disorder	Plasma (P2–1)	*NRAS* Q61R	0.20	41,269	0.07	0.13	0.02
					*NRAS* WT	275.93	41,269	–	–	–
				PE (P2–2)	*NRAS* Q61R	0.03	39,120	0.16	0.55	0.02
					*NRAS* WT	18.28	39,120	–	–	–
				FFPE tissue^f^ (P2–3)	*NRAS* Q61R	0.14	33,434	0.2	0.4	0.00
					*NRAS* WT	70.7	33,434	–		
P3 (KLA)	4/ F	Thoracic and mediastinal	Hemorrhagic PE and coagulation disorder	Plasma (P3–1)	*NRAS* Q61R	0.06	37,013	0.02	0.06	0.00
					*NRAS* WT	280.55	37,013	–	–	–
				PE (P3–2)	*NRAS* Q61R	0.03	38,740	0.01	0.04	0.00
					*NRAS* WT	229.55	38,740	–	–	–
P4 (KLA)	12/ M	Bone, thoracic and mediastinal	Scoliosis, hemorrhagic PE and coagulation disorder	Plasma (P4)	*NRAS* Q61R	0.06	41,040	0.02	0.06	0.00
					*NRAS* WT	243.08	41,040	–	–	–
P5 (KLA)	20/ M	Bone, thoracic and mediastinal	PE and coagulation disorder	Plasma (P5)	*NRAS* Q61R	0.00	37,721	0.00	0.00	0.00
					*NRAS* WT	27.52	37,721	–	–	–
P6 (GLA)	18/ M	Bone, thoracic and mediastinal	PE and coagulation disorder	Plasma (P6)	*NRAS* Q61R	0.06	39,832	0.03	0.07	0.00
					*NRAS* WT	226.91	39,832	–	–	–
P7 (GLA)	35/ F	Abdominal cavity and skin	Lymphorrhea, pain, and cellulitis	Plasma (P7)	*NRAS* Q61R	0.00	36,076	0.00	0.00	0.00
					*NRAS* WT	39.09	36,076	–	–	–
P8 (GLA)	32/ F	Abdominal cavity and skin	Ascites, coagulation disorder, and lymphorrhea	Plasma (P8)	*NRAS* Q61R	0.00	42,470	0.00	0.00	0.00
					*NRAS* WT	49.65	42,470	–	–	–
Positive control				Plasma (PC)	*NRAS* Q61R	0.00	13,194	0.00	0.00	0.00
					*NRAS* WT	352.38	13,194	–	–	–
Negative control				(NC)	*NRAS* Q61R	0.00	12,603	0.00	0.00	0.00
					*NRAS* WT	0.00	12,603	–	–	–
Human skin melanoma cell lines (SK-MEL-2)^g^					*NRAS* Q61R	106.16	34,258	69.15	70.52	67.77
					*NRAS* WT	47.37	34,258	–	–	–

Characteristics (diagnosis, age, sex, affected organs, and complications) of the patients with KLA (P1–5) and GLA (P6–8) are shown in the table. The information of the samples (sample materials) and results (concentration of target gene, accepted droplets, fractional abundance and percentage of Poisson fractional abundance) from a droplet digital PCR experiment on *NRAS* p.Q61R mutations are shown. Positive controls included wild-type DNA. Negative controls did not include DNA. Human melanoma cell line with *NRAS* p.Q61R mutation was used as a positive control for *NRAS* mutation

*KLA* kaposiform lymphangiomatosis, *GLA* generalized lymphatic anomaly, *P* patient, *PC* positive control, *NC* negative control, *M* male, *F* female, *PE* pleural effusion, *WT* wild type, *FFPE* formalin-fixed, paraffin-embedded

^a^Target gene copy number per microliter

^b^Number of droplets measured—i.e., the sum of three measured wells

^c^Percentage of mutant droplets relative to the sum of wild-type droplets. It was statistically calculated from the measurements of three wells

^d^Percentage of Poisson fractional abundance (upper limit of the 95% confidence interval)

^e^Percentage of Poisson fractional abundance (lower limit of the 95% confidence interval)

^f^FFPE tissue of the affected lesion in P2

^g^*NRAS* Q61R mutated sample was the positive control

### WES and cfDNA analysis in patient 1

**Fig. 2:**
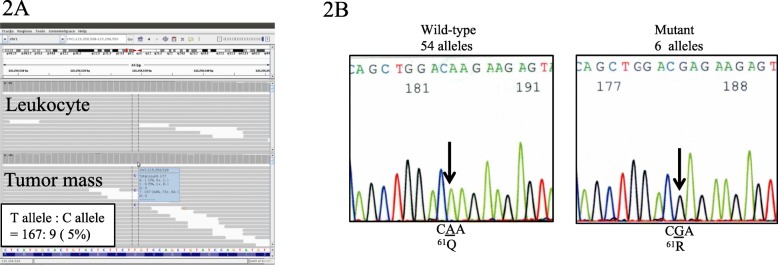
Results of whole-exome sequencing and Sanger sequencing of the patient with KLA (P1). **a** Integrative Genomics Viewer (IGV) view of *NRAS* c.182G > A in DNA samples from leukocytes (upper) and tumor mass (lower). c.182G > A allele was detected in 9 of 176 (5%) alleles. IGV shows reverse strands. **b** Results of Sanger sequencing of pTA cloning vectors. *NRAS* exon 2 was amplified by PCR in tumor DNA sample as a template. PCR product was subcloned into pTOPO cloning vector and each plasmid was sequenced. Six of sixty clones (10%) had a c.182G > A allele

**Fig. 3:**
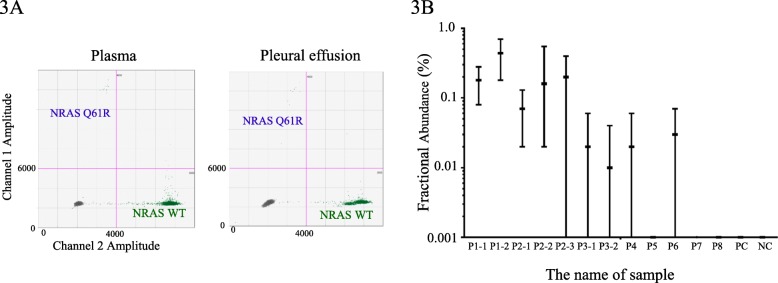
Cell-free DNA analysis of GLA and KLA patients. **a** Gene multiplexing on a droplet digital polymerase chain reaction (ddPCR) system using probes that target specific *NRAS* mutation in cell-free DNA isolated from plasma and pleural effusion samples (P1). *NRAS* mutation detection assays were performed using the mutation detection assay *NRAS* p.Q61R c.182A > G (Bio-Rad) in a ddPCR apparatus (QX200™ AutoDG™ Droplet Digital™ PCR system; Bio-Rad). Two fluorescence amplitude bands were clearly generated for each gene (upper band: *NRAS* Q61R mutant, lower band: *NRAS* wild type). **b** The data are expressed as a percentage of mutant droplets relative to the sum of wild-type droplets of each sample (P1–8, positive control and negative control). Positive controls included wild-type DNA. Negative control did not include DNA. Data are shown as the median and 95% confidence interval. The identities of the samples are as follows: P1–1: plasma sample of P1, P1–2: PE sample of P1, P2–1: plasma sample of P2, P2–2: PE sample of P2, P2–3: FFPE sample of P2, P3–1: plasma sample of P3, P3–2: PE sample of P3, P4–8: plasma sample of P4–8, PC: plasma sample of PC, NC: plasma sample of NC. WT: wild type, PE: pleural effusion, PC: positive control, NC: negative control

WES analysis was performed on DNA from leukocytes and biopsy specimens from the chest wall mass of patient 1 (P1). We first compared the variants between the tumor samples and the blood samples using data from the BWA-GATK pipeline. No germline or somatic variants that could be associated with lymphatic abnormalities were identified. We then filtered less frequent variants that were specifically identified in the chest mass samples using MuTect software. Of those, the *NRAS* c.182A > G variant (p.Q61R) was identified in 9/176 alleles (5%) of the tumor samples, while no mutant reads were detected in the leukocyte samples (Fig. 2a). We then performed PCR using primers that amplify exon 2 of *NRAS*. Subcloning and Sanger sequencing of the PCR product identified the c.182A > G variant in 6 of 60 clones (10%) (Fig. 2b). These results suggested the presence of a low-frequency somatic *NRAS* variant in tumor tissues. Furthermore, the cfDNA was extracted from the plasma (5 ml) and pleural effusion (5 ml) (790.4 ng and 93.8 ng, respectively). *NRAS* c.182A > G mutation was detected at a low frequency in cfDNA isolated from plasma and pleural effusion using ddPCR (Fig. 3a, b and Table 1).

### cfDNA analysis in plasma and pleural effusions from other KLA and GLA patients

We performed cfDNA analysis in the plasma and pleural effusion of KLA (P2–5) and GLA patients (P6–8). We obtained pleural effusion samples from P2 and P3 (P2–2 and P3–2). In cfDNA analysis, a low-frequency *NRAS* p.Q61R mutation was detected in both plasma and pleural effusion samples of P2 and P3 (P2–1, P2–2, P3–1, and P3–2) and plasma samples of P4 and P6. Additionally, a low-frequency *NRAS* p.Q61R mutation was detected in FFPE tissue of the affected bone lesion in P2 (P2–3) (Fig. 3b and Table 1). P3–8 had not undergone genetic analysis of the lesions to determine whether the same *NRAS* mutation could be detected from these samples.

## Discussion

We here present the first study of liquid biopsy for *NRAS* mutation in KLA cases. In a first case with intractable KLA treated with sirolimus, which induced improvement of symptoms and tumor regression, WES of DNA samples from leukocytes and tumor tissues showed the presence of *NRAS* c.182A > G (p.Q61R) in 5% of alleles in tumor tissues, which was confirmed by TA cloning of the PCR product followed by sequencing. The *NRAS* mutation was also detected in cfDNA isolated from plasma and pleural effusion. Furthermore, we conducted target-specific *NRAS* mutation analysis by using cfDNA isolated from liquid sample of 7 patients (4KLA and 3GLA) and detected the targeted gene mutation in 80% (4/5) of KLA patients (P1, P2, P3, and P4). The genotyping of FFPE tissue showed that the same mutation was also detected in an affected lesion from a KLA patient (P2). These results suggested that this somatic oncogenic *NRAS* mutation may be involved in the pathogenesis of KLA, and cfDNA might be useful for diagnosing KLA.

KLA is recognized as a new entity that has an aggressive course and poor prognosis. Recent research has attempted to elucidate the actual conditions and pathogenesis of these diseases. KLA was classified by the World Health Organization as a rare tumor of lymphatic vessel origin with the capacity to metastasize [10]. Although the ISSVA classification of 2014 categorized this condition as a provisionally unclassified vascular anomaly, KLA was categorized as a subtype of GLA in the ISSVA classification updated in 2018 [2]. The key signals of KLA are thrombocytopenia, coagulation disorder, and hemorrhagic pericardial and pleural effusion or ascites. However, the pathogenesis and etiology of KLA are still unknown. GLA also involves the diffuse or multicentric proliferation of lymphatic vessels in several organ systems, and an appropriate diagnosis is difficult because the clinical findings of KLA and GLA overlap [1]. Dilated malformed lymphatic channels lined by a single layer of endothelial cells are common to both GLA and KLA; the latter also has foci of patternless clusters of intra- or peri-lymphatic spindle cells associated with platelet microthrombi, extravasated red blood cells, hemosiderin, and some degree of fibrosis. Recently, two reports on cytokine analysis in patients with lymphatic anomalies showed that angiopoietin-2 and some cytokines are important markers for KLA [11, 12]. However, differentiation of these diseases is challenging based on their phenotypic presentation alone, so further study is needed.

Although genetic analysis revealed somatic mutations in genes associated with the phosphoinositide 3-kinase (PI3K) pathway in patients with LAs [4], little has been reported on the associated genetic abnormalities of KLA. In a recent study, Manevitz-Mendelson et al. reported the possibility that somatic *NRAS* mutation causes GLA [6]. Activating mutations in *RAS* proto-oncogenes (*KRAS*, *HRAS*, and *NRAS*) have been found in a variety of human malignancies, suggesting a dominant role in carcinogenesis [13–15]. The authors isolated lymphangiomatosis endothelial cells from a GLA patient using CD31-coated magnetic beads and identified a somatic activating mutation in *NRAS* in fewer than 30% of the alleles of the endothelial cells. The results of the study showed that the *NRAS* mutation plays key roles in the regulation of angiogenesis and lymphangiogenesis. Barclay et al. reported that *NRAS* mutations were detected in the affected lesions of KLA patients [5]. We also found that KLA patients possessed the same *NRAS* mutation. Although why the same *NRAS* mutation could be associated with both GLA and KLA is not clear, it could be speculated that the confusion is due to not only the biology of these diseases but also the difficulty of the diagnosis. The pathogenesis of these diseases requires further investigation.

In a wide spectrum of RAS-related disorders, recently called RASopathies [16], somatic *RAS* mutations, especially somatic *NRAS* mutation at codon 61 (p.Q61R/K), have been identified in a variety of human malignancies [15]. Somatic *NRAS* c.182A > G (p.Q61R) mutations have also been identified in nonmalignant cancers, including pyogenic granuloma [17] and Langerhans cell histiocytosis [18]. In contrast, germline *NRAS* mutations have been identified in patients with Noonan syndrome, which is characterized by short stature, congenital heart disease, lymphatic abnormalities, chest deformity, and predisposition to malignant tumors [16]. However, *NRAS* p.Q61R mutation has never been identified in Noonan syndrome patients, suggesting that patients with germline p.Q61R mutation do not survive to birth because of its strong activation of the downstream pathway. In mosaic RASopathies, *NRAS* p.Q61R mutations have been identified in a patient with Schimmelpenning syndrome [19], patients with neurocutaneous melanosis [20], and patients with cutaneous–skeletal hypophosphatemia syndrome [21]. These results suggest that somatic or mosaic *NRAS* p.Q61R mutations cause a broad spectrum of disorders, which depend on the lineages or cells in which they occur.

Low-frequency *NRAS* mutation was identified in a chest mass in our patient. However, we were not able to identify in which cells this mutation arose. *NRAS* mutations play a critical role in angiogenesis and lymphangiogenesis [6]. We also performed ddPCR assays of the *NRAS* p.Q61R mutation for other KLA and GLA patients who had not undergone WES (genetic) analysis because we did not have fresh frozen samples of their affected lesions. A total of 80% (4/5) of patients with KLA and 33.3% (1/3) of patients with GLA showed low-frequency *NRAS* mutation in cfDNA isolated from plasma or pleural effusion. Additionally, the same mutation was detected in the FFPE tissue of another KLA patient (P2). Although the results of P3, 4, and 6 could be false positives because the minimum Poisson fractional abundance in their samples was statistically 0.0, the *NRAS* p.Q61R mutation was apparently detected in tissue samples from two KLA patients (P1 and P2). Our results indicate the possibility of developing a novel diagnostic method, a so-called liquid biopsy, for KLA without the need for an invasive procedure.

The finding that *RAS* mutation drives vascular anomalies including GLA and KLA provides potential opportunities to develop targeted therapies for current drug-resistant lesions. Treatment with an mTOR inhibitor, sirolimus, and an MEK inhibitor, trametinib, had an effect of reducing the viability of the affected cells through inhibition of the phosphorylation of AKT and extracellular signal-regulated kinase (ERK) [6]. *NRAS* inhibition by these drugs might be a promising option for the treatment of these lymphatic anomalies. In our patient, sirolimus was effective to improve clinical symptoms and subcutaneous lesions. These genes are associated with the pathogenic etiology of lymphatic diseases and their inhibition might thus be a target for treatment.

## Conclusions

Our results provide the first evidence of an *NRAS* oncogenic variant in cfDNA in two patients with KLA. Furthermore, this is the first time that mutated gene from isolated cfDNA has been detected in patients with vascular anomalies. The observation that KLA can be caused by mutations in *NRAS* offers insight into the basic biology of KLA. It suggests that KLA is a group of RASopathies, which may facilitate efforts to develop an appropriate therapy for it. Furthermore, liquid biopsy using cfDNA could be a new useful method for these diseases.

## Data Availability

The datasets and analysis performed during the current study are available from the corresponding author upon reasonable request.

## Abbreviations

BWA: Burrows–Wheeler Alignment
cfDNA: Cell-free DNA
ddPCR: Droplet digital polymerase chain reaction
ERK: Extracellular signal-regulated kinase
ExAC: Exome Aggregation Consortium
FFPE: Formalin-fixed, paraffin-embedded
GATK: Genome Analysis Toolkit
GLA: Generalized lymphatic anomaly
HGVD: Human Genome Variation Database
IGV: Integrative Genomics Viewer
iJGVD: Integrative Japanese Genome Variation Database
ISSVA: International Society for the Study of Vascular Anomalies
KLA: Kaposiform lymphangiomatosis
LA: Lymphatic anomaly
MRI: Magnetic resonance imaging
mTOR: Mammalian target of rapamycin
P: Patient
PI3K: Phosphoinositide 3-kinase
WES: Whole-exome sequencing
